# Antimicrobial Resistance Profile of Different Clinical Isolates against Third-Generation Cephalosporins

**DOI:** 10.1155/2018/5070742

**Published:** 2018-09-09

**Authors:** Fanta Gashe, Eshetu Mulisa, Mekidim Mekonnen, Gemechu Zeleke

**Affiliations:** ^1^School of Pharmacy, Institute of Health, Jimma University, P.O. Box 378, Jimma, Ethiopia; ^2^School of Laboratory Sciences, Institute of Health, Jimma University, P.O. Box 378, Jimma, Ethiopia

## Abstract

**Background:**

Drug resistant microorganisms lead to an increase in morbidity and mortality as they boost the risk of inappropriate therapy. Hence, data on antimicrobial resistance help define the best possible treatment for individual patients. Therefore, this study aimed to screen the antimicrobial resistant profile of 3rd generation cephalosporin drugs in Jimma University Specialized Teaching Hospital.

**Methods:**

A hospital based prospective cross-sectional study was conducted in Jimma University Specialized Hospital (JUSH) from April to August 2016. The clinical samples such as wound swab, urine, sputum, and stool were collected from hospitalized patients. Then, bacterial species were isolated and identified as per the standard microbiological methods. Antimicrobial susceptibility tests were carried out using various antimicrobial discs by Kirby–Bauer disc diffusion method.

**Results:**

Totally, 248 bacterial isolates were obtained from 154 (62.1%) male and 94 (37.9%) female patients.* Escherichia coli* (25.4%) and* Staphylococcus aureus* (19.0 %) were the predominant organisms isolated from specimens. About 140 (56.5%) and 149 (60.1%) of the total bacterial isolates were found to be resistant to ceftriaxone and ceftazidime, respectively. The majority of* Escherichia coli* isolates 46 (73%) were resistant to ceftriaxone and 41 (65%) of them were resistant to ceftazidime.* Staphylococcus aureus, *which accounted 19% of the total bacterial isolates, showed 23.4% and 34% resistance to ceftriaxone and ceftazidime, respectively. Among the bacterial strains revealing resistant to ceftriazone and ceftazidime, about 109 (44%) and 108 (43.5%) of them were resistant to two, three, or four other drugs, respectively.

**Conclusion:**

Bacterial resistance towards third-generation cephalosporin (ceftriaxone and ceftazidime) is escalating as more than half of the isolated strains demonstrated resistance to these drugs. Moreover, these strains also revealed multidrug resistance mainly against clinically used drugs which could render therapy unsuccessful. Therefore, in clinical use appropriate medications should be selected based on the data obtained from antimicrobial susceptibility tests.

## 1. Introduction

Antimicrobial resistance (AMR) is a growing problem in the 21^st^ century and one of the most serious jeopardies to global public health [1]. The number of resistant microbial strains, geographic areas affected by drug resistance, and the extent of resistance in each organism are escalating [2]. Moreover, the percentages of organisms exhibiting AMR, especially resistance to multiple antibiotics, are continuingly increased [3]. Thus, disease agents that were once thought to be susceptible to antibiotics are returning in new leagues resistant to these therapies [4].

Resistant microorganisms lead to an increase in morbidity and mortality since it increases the risk of inappropriate therapy [5, 6]. This resistance may delay and hinder treatment, resulting in complications or even death [7, 8]. Moreover, a patient may need more care, as well as the use of alternative and more expensive antibiotics, which may have more severe side effects or may need more invasive treatments, such as intravenous injection, to be given in hospitals [6, 9].

Multiply resistant organisms render therapy more precarious and costly and sometimes unsuccessful. Individuals may succumb to multidrug resistance (MDR) infections because all available drugs have failed, especially in the developing world [10]. For instance, MDR enteric disease agents have threatened public health in developing countries [3]. Globally, MDR were reported in* Mycobacterium tuberculosis*,* Enterococcus faecium*,* Enterobacter cloacae*,* Klebsiella pneumoniae*,* Staphylococcus aureus*,* Acinetobacter baumanii, *and* Pseudomonas aeruginosa* [11].

Historically, many infections could be treated successfully according to the clinician's past clinical experience (i.e., empirical therapy) [12, 13]. However, this practice is becoming more than the exception to the rule since resistance has been observed to essentially all of the antimicrobial agents currently approved for use in human and veterinary clinical medicine. This, combined with the variety of antimicrobial agents currently available, makes the selection of an appropriate agent an increasingly more challenging task. Hence, this situation has made clinicians more dependent on data from* in vitro* antimicrobial susceptibility testing and highlights the importance of the diagnostic laboratory in clinical practice [14].

Data on AMR among local pathogens help define the best possible treatment for individual patients [15, 16]. However, the proportion of resistant bacteria can vary from one area to another [17], and in many health facilities there are no local data on resistance patterns [18]. Experiences from surveillance networks on antimicrobial use and AMR show that data, where available, can be put to multiple uses, including orienting treatment choices, understanding AMR trends, informing public health policy, identifying priority areas for interventions, and monitoring the impact of interventions to contain resistance [1]. However, there is no sufficient data on antimicrobial resistance profile of antibiotics especially in developing country like Ethiopia. Therefore, the present study involves the screening of the antimicrobial resistant profile of 3^rd^ generation cephalosporin drugs that are used in the treatment of infectious diseases in Jimma University Specialized Teaching Hospital.

## 2. Materials and Methods

### 2.1. Study Design and Specimen Collection

A hospital based cross-sectional study was conducted in Jimma University Specialized Hospital (JUSH) from April to August 2016. The hospital was selected because of the diverse services it provides for wide range of health problems of the patients who come from different parts of the country. The clinical samples such as wound swab, urine, sputum, and stool were collected from hospitalized patients by trained nurses.

### 2.2. Bacteria Identification

For the detection and isolation of pathogenic bacteria, all the clinical samples were collected by standard microbiological technique. Then, depending on the source of samples, each specimen was platted onto MacConkey agar, Blood agar, Mannitol Salt agar, Xylose lysine deoxycholate agar, Chocolate agar, and Thayer–Martin agar and then incubated aerobically at 37°C for 24 h.

Gram-positive cocci in cluster, both catalase and coagulase positivity, and characteristically yellow to golden colored colonies on blood agar coupled with mannitol fermentation on MSA were applied to identify* Staphylococcus aureus* from other gram-positive cocci. The gram-negative bacilli, the coliforms,* Proteus *spp.,* and Yersinia enterocolitica *were identified by standard microbiological algorisms such as gram's stain (gram-negative bipolarly stained bacilli for* Yersinia *spp) colonial growth characteristics and appearance on enriched and selective media indicated combined with standard biochemical tests outlined in the reference material [19]. Biochemical tests such as fermentation of lactose, glucose, and sucrose with and without H_2_S production (using TSI/KIA); lysine decarboxylation (LDC); indole and citrate utilization (MIS); methyl red (MR), Voges-Proskauer (VP); and pyrrolidonyl aminopeptidase (PYR) were used to identify the clinical isolates in question and of clinical significance [19, 20]. Thus, clinical strains of* Staphylococcus aureus, Escherichia Coli, Klebsiella pneumoniae, Proteus *species,* Citrobacter freundii, Citrobacter Koseri, Enterobacter cloacae, Klebsiella oxytoca, Enterobacter aerogenes,* and* Yersinia enterocolitica *were isolated from the collected clinical samples.

### 2.3. Antimicrobial Susceptibility Testing

Antimicrobial susceptibility testing was done using disk diffusion technique according to Kirby–Bauer method using* S. aureus*  ATCC 25923 and as quality control strains [21]. Accordingly, at least three to five well-isolated colonies of the same morphological type were selected from an agar plate culture and transferred into Muller Hinton broth and incubated at 37°C for 24 hours. The turbidity of the suspension was adjusted with sterile saline to obtain turbidity optically comparable to that of the 0.5 McFarland standards. Then, the swab was streaked over the entire surface of the freshly prepared Mueller Hinton agar plate. The antimicrobial disks were applied to the plates within 15 minutes after inoculation. The plates were then incubated at 37°C for 24 hours. A zone of inhibition was measured and the results were interpreted as sensitive, resistant, or intermediate based on resistance data interpreted according to Clinical and Laboratory Standards Institute [22]. The antimicrobial agents tested were third-generation cephalosporin: ceftriaxone (30 *μ*g) and ceftazidime (CAZ) (30 *μ*g). Moreover, MDR profile for those strains resistant to cephalosporin drugs were determined against different classes of antimicrobials such as ciprofloxacin (5 *μ*g), sulfamethoxazole-trimethoprim (25 *μ*g), amikacin (AMK) (30 *μ*g), piperacillin (PIP) (100 *μ*g), Amox-clavulanic acid (AUG), and ciprofloxacin (CPR) (5*μ*g). All the antibiotic discs used were manufactured by Abtek Biologicals Ltd., Liverpool L9 7AR, UK.

### 2.4. Quality Control

The reliability of the study findings was guaranteed by implementing quality control measures throughout the whole process of the laboratory work. Staining reagents, culture medias, and antibiotic discs were checked for their normal shelf life before use. All culture plates and antibiotic discs were stored at recommended refrigeration temperature after being prepared and sterilized by autoclaving at 121°C for 15 minutes. The standard reference bacterial strains were tested as a positive control on the biochemical tests and agar plates with antibiotic discs. Proper sample collection and handling were done by experienced nurses who were working at each ward unit.

### 2.5. Data Analysis

Data were edited, cleaned, entered, and analyzed using statistical package for social science (SPSS) version 16. Descriptive analysis such as frequencies and mean were used. P value of < 0.05 was considered to indicate statistically significant differences and the results were presented using tables and figure.

## 3. Results

About 388 clinical specimens were collected from sputum, urine, wound swab, and stool of hospitalized patients having clinically evident infection (patients with complaints of urinary tract infection, open wounds, pneumonia, and upper respiratory tract infections). Totally, 248 (64%) bacterial isolates were obtained from 154 (62.1%) male and 94 (37.9%) female study subjects. In the present study* Escherichia coli* (25.4%) and* Staphylococcus aureus* (19.0 %) were the predominant organisms isolated from the study subjects. The other bacterial isolates include* Citrobacter freundii *(12.1%)*, Citrobacter koseri *(8.5%)*, Enterobacter cloacae *(13.0%),* Klebsiella oxytoca *(2.4%),* Klebsiella pneumoniae *(10.5%)*, Enterobacter aerogenes *(4.8%)*, Proteus *species (2.0%), and* Yersinia enterocolitica *(2.4%) as indicated in Table 1.

**Figure 1:**
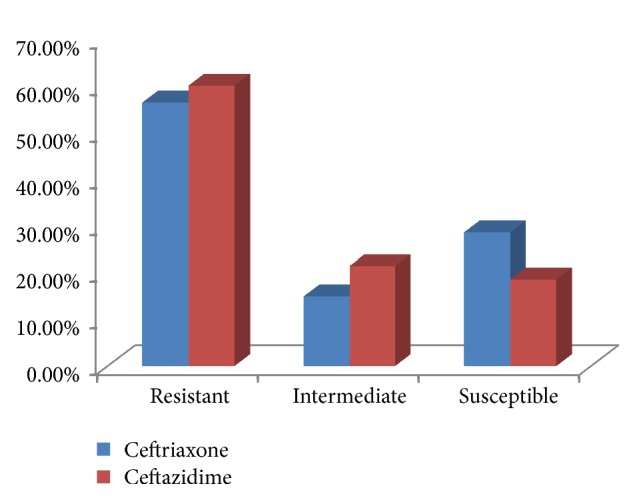
Resistance profile of clinical isolates to ceftriaxone and ceftazidime.

All the bacterial isolates were tested for susceptibility against selected third-generation cephalosporins (ceftriaxone and ceftazidime). Out of 248 bacterial isolates, 140 (56.5%) were found to be resistant to ceftriaxone. But, 37 (14.9%) and 71 (28.6%) of the isolates remain intermediate and susceptible to ceftriaxone, respectively. On the other hand, 149 (60.1%) of the total bacterial isolates were found to be resistant, 53 (21.4%) were intermediate, and only 46 (18.5%) were susceptible to ceftazidime (Figure 1).

As shown in Tables 3 and 4, the rate of bacterial isolates resistant to ceftriaxone and ceftazidime was 56.5% and 60.1%, respectively. Majority of the urinary tract isolates were found to be resistant to the action of third-generation cephalosporins (ceftriaxone or ceftazidime). Out of 63* Escherichia coli* isolates, 46 (73%) were resistant to ceftriaxone which is very high. Moreover, about 41 (65%) of them were resistant to ceftazidime.* Citrobacter freundii, *which is another urinary pathogen, showed a resistance of 36.7% (11/30) to ceftriaxone and 43.3% (13/30) to ceftazidime.

In this study, most of the Enterobacteriaceae (*Citrobacter koseri*,* Enterobacter cloacae*,* Klebsiella oxytoca*,* Enterobacter aerogenes, *and* Proteus* species) isolates were resistant to ceftriaxone or ceftazidime. In addition,* Staphylococcus aureus*, which accounted 19% of total bacterial isolates, showed 23.4% (11/47) and 34% (16/47) resistance to ceftriaxone and ceftazidime, respectively. Similarly,* Klebsiella pneumoniae *showed 46.1% (12/26) resistance to ceftriaxone. More than 90% (10/11) of* Enterobacter aerogenes* were resistant to ceftazidime and none of the* Proteus* species were susceptible to the action of ceftriaxone or ceftazidime.

The multidrug resistance pattern showed that among the bacterial strains found to be resistant to ceftriazone and ceftazidime about 109 (44%) and 108 (43.5%) were resistant to two, three, or four drugs, respectively.* Escherichia coli, Staphylococcus aureus, Enterobacter* species, and* Citrobacter* species showed resistance to two, three, or four drugs. On the other hand,* Citrobacter* species and* Proteus* species were resistant to two or three drugs while* Klebsiella Pneumonia* revealed resistance to two drugs.

## 4. Discussion

The widespread use of brood spectrum antibiotics has led to the emergence of antibiotic resistant strains of bacteria. High rates of resistance have been primarily observed in bacteria that cause common health problems. In the present study more than half of the isolated bacteria strains were resistant to either ceftriaxone or ceftazidime drugs which is in agreement with 2014 WHO reports [1].

The drug resistance pattern differences among isolates based on various characteristics were evaluated (Table 2). In view of that, there were no significant differences observed except for the specimen types from which the strains were isolated. Most of the urinary tract isolates were found to be resistant to the action of third-generation cephalosporins (ceftriaxone or ceftazidime). The majority of these isolates were* Escherichia coli* which is a gram-negative bacterium. This uropathogen is the major extended spectrum beta-lactamase (ESBL) producer, severely limiting the therapeutic management in cases of urinary tract infections [23]. Hence, isolates of these strains have relatively high potentials of developing resistance [12].

Moreover, most of* Escherichia coli* strains isolated from the whole specimen were found to be resistant to the action of ceftriaxone and ceftazidime in the present study. It was also revealed that the proportion of resistance to third-generation cephalosporins increased significantly for* Escherichia coli* infections since 2004 [24]. Similarly, other research finding reported that* Escherichia coli* exhibited the highest resistance to ceftazidime and ceftriaxone [25, 26]. However, the study in University of Gondar Hospital, Ethiopia, showed that the percentage of resistance strains observed against ceftazidime was high but relatively less to ceftriaxone [27].

The majority of* Klebsiella pneumoniae* strains were more resistant to ceftazidime compared to ceftriazone in this study. However, it is dissimilar with other studies which showed that the isolates exhibited similar resistance pattern to both ceftazidime and ceftriaxone [28, 29]. It was also reported that* Klebsiella pneumoniae* strain isolated from patients with community acquired pneumonia was resistant to third-generation cephalosporins [30, 31]. This is because these strains have a *β*-lactam ring provided with a Zwitterionic structure that protects these antibiotics from hydrolysis by *β*-lactamases [32]. On the contrary, the study conducted in Oman stated that most of the isolated strains were susceptible towards third-generation cephalosporin-ceftriaxone [33].

*Staphylococcus aureus *strains were found to be more susceptible than other bacteria strains to ceftriaxone and ceftazidime which is inconsistent with previous study in which most of the strains were resistant [34]. However, it is in line with other studies conducted in different areas which reported the susceptibility of the strains towards the third-generation cephalosporins [33, 35, 36]. On the other hand, in the study carried out in Dessie Hospital, Ethiopia, the resistance pattern for clinical isolate against ceftriazone was about 43.5% which is more than the present study. These findings indicate that the resistance rate of* Staphylococcus aureus *varies from area to area or/and period to period even within the same country.

Most of the Enterobacteriaceae (*Citrobacter koseri, Enterobacter cloacae, Klebsiella oxytoca*,* Enterobacter aerogenes, *and* Proteus *species) tested isolates were resistant to ceftriaxone or ceftazidime. Similarly,* in vitro* antimicrobial study in Senegal revealed that most of the isolated Enterobacteriaceae strains were resistant to third-generation cephalosporins [36]. On the other hand, it was reported that* Enterobacter* species were relatively more resistant to ceftriaxone than ceftazidime [37]. Similar resistance pattern with present study was reported for* Enterobacter cloacae *against ceftriazone [38].

Multidrug resistance pattern of isolated strains, which were found to be resistant to either of ceftriaxone and ceftazidime, was also evaluated. The majority of* Escherichia coli* and* Staphylococcus aureus* strains exhibited resistance against two, three, or four antimicrobials. About half of* Escherichia coli* strains resistant to third-generation cephalosporins were also resistant to clinically used drugs such as amikacin, sulfamethoxazole-trimethoprim, piperacillin, and ciprofloxacin. This could be due to the high rate of adaptive mutation. Resistant organisms transfer their resistant genes either to their offspring by replication (vertical gene transfer) or by conjugation where the plasmids carrying the resistant gene are exchanged between the nearby organisms (horizontal gene transfer) [1, 39].

## 5. Conclusion

Microbial resistance to third-generation cephalosporin drugs have been increasing significantly as the finding of the present study indicated. Moreover, those strains which developed resistance to third-generation cephalosporins were also resistant to multiple drugs which could make treatment of infectious disease triggered by these microbial strains become challenging (Table 5). Therefore, the right medications should be selected based on susceptibility data of causative agents towards the drugs for the treatment of right disease agents.

## Figures and Tables

**Table 1:** Distribution of isolates in clinical specimens collected from patients.

**Clinical isolates**	**Specimen type**				
	Sputum	Urine	Wound Swab	Stool	**Total**
*Escherichia coli*	-	29	3	31	63
*Citrobacter *spp.	3	11	25	12	51
*Enterobacter species*	16	13	15	-	44
*Klebsiella oxytoca*	-	-	6	-	6
*Klebsiella pneumonia*	23	-	-	3	26
*Staphylococcus aureus*	6	3	38	-	47
*Proteus species*	-	-	5	-	5
*Yersinia enterocolitica*	3	-	3	-	3
**Total**	51	56	95	46	248

**Table 2:** Sociodemographic characteristics association with resistance pattern of clinical isolates.

**Characteristics**		**Ceftazidime **			**Ceftriaxone**		
		R	NR	P-value	R	NR	P-value
**Age in years**	≤19	23	20	0.07622	23	20	0.06902
	20-64	80	61		73	68	
	≥65	46	18		44	20	

**Sex**	Female	55	39	0.69326	53	41	0.98641
	Male	94	60		87	67	

**Specimen Type**	Sputum	29	22	0.08527	26	25	0.01426
	Urine	41	15		41	15	
	Wound Swab	50	45		45	50	
	Stool	29	17		28	18	

**Hospital Stay**	≤1 Days	35	27	0.29227	30	32	0.35481
	2-3 Days	72	40		66	46	
	4-6 Days	19	20		21	18	
	≥7 Days	23	12		23	12	

**Table 3:** Resistance pattern of the different clinical isolates to ceftriaxone.

**Clinical isolates**	**Resistance pattern**			
	**Resistant**	**Intermediate**	**Susceptible**	**Total**
*Citrobacter species*	27(52.9%)	13(25.5%)	11(21.6%)	**51**
*E. coli*	46 (73.0%)	3 (4.8%)	14 (22.2%)	**61**
*Enterobacter species*	31 (70.4)	5(11.4%)	8(18.2%)	**44**
*K. pneumonia*	12 (46.2%)	4 (15.4%)	10 (38.4%)	**26**
*K. oxytoca*	5 (83.3%)	0	1 (16.7%)	**6**
*S. aureus*	11 (4.4%)	10(4.0%)	26(10.5%)	**47**
*Proteus species*	4 (80%)	1 (20%)	0	**5**
*Y. enterocolitica*	4 (66.6%)	1 (16.7%)	1 (16.7%)	**6**
**Total**	**140 (56.5)**	**37 (14.9%)**	**1 (16.7%)**	**248 (100)**

**Table 4:** Resistance pattern of the different clinical isolates to ceftazidime.

**Clinical isolates**	**Resistance pattern**			
	**Resistant**	**Intermediate**	**Susceptible**	**Total**
*E. coli*	41 (65.1%)	10 (15.9%)	12 (19.0%)	**63**
*Citrobacter species*	29 (56.9%)	8 (15.7%)	14 (27.4%)	**51**
*S. aureus*	16 (34.0%)	20 (42.6%)	11 (23.4%)	**47**
*Enterobacter species*	35 (79.6%)	6 (13.6%)	3(6.8%)	**44**
*K. pneumonia*	19 (73.1%)	5 (19.2%)	2 (7.7%)	**26**
*K. oxytoca*	4 (80%)	2 (20%)	0	**6**
*Y. enterocolitica*	2 (33.3%)	0	4 (66.7%)	**6**
*Proteus species*	3 (60.0%)	2 (40.0%)	0	**5**
**Total**	**149 (60.1%)**	**53 (21.4%)**	**46 (18.5%)**	**248 (100)**

**Table 5:** Multidrug resistance pattern of microbial strains.

**Clinical Isolates**	**Multi-drug resistance pattern**			
	**Resistance **	**Number of isolates**	**Resistance **	**Number of isolates**
***Escherichia coli(n=63)***	CTR only	46	CAZ only	41
	CTR, SXT	42	CAZ+SXT	36
	CTR,SXT,AUG	21	CAZ+SXT+AUG	20
	CTR,SXT,AUG,CPR	20	CAZ,SXT,AUG,CPR	19

***Klebsiella Pneumonia (n=26)***	CTR only	12	CAZ only	19
	CTR,CPR	3	CAZ,CPR	3
	CTR,CPR,AMK	0	CAZ,CPR,AMK	0

***Staphylococcus aureus (n=47)***	CTR only	11	CAZ only	16
	CTR,CPR	6	CAZ,CPR	7
	CTR,CPR,AUG	2	CAZ,CPR,AUG	2

***Citrobacter species*** ***(n=51)***	CTR only	27	CAZ only	29
	CTR,PIP	25	CAZ,PIP	27
	CTR,PIP,CPR	9	CAZ,PIP,CPR	9
	CTR,PIP,CPR,AMK	1	CAZ,PIP,CPR,AMK	0

***Enterobacter species*** ***(n=44)***	CTR only	32	CAZ only	36
	CTR,PIP	30	CAZ,PIP	33
	CTR,PIP,CPR	13	CAZ,PIP,CPR	14
	CTR,PIP,CPR,AMK	1	CAZ,PIP,CPR,AMK	1

***Proteus species*** ***(n=5)***	CTR only	4	CAZ only	3
	CTR+PIP	3	CAZ+PIP	2
	CTR,PIP,CPR	1	CAZ+PIP+CPR	1
	CTR,PIP,CPR,AMK	0	CAZ,PIP,CPR,AMK	0

CTR= ceftriaxone, SXT = sulfamethoxazole-trimethoprim, AMK= amikacin,, PIP= piperacillin, CAZ= ceftazidime,, AUG= Amox-clavulanic acid, and CPR= ciprofloxacin.

## Data Availability

Complete organized and compiled research data were included in this paper and a complete dataset will be available from the corresponding author on request.

## References

[1] World Health Organization (2000). *Essential Drugs Monitor: Antimicrobial Drug Resistance: A Global Threat*.

[2] Pfeifer Y., Cullik A., Witte W. (2010). Resistance to cephalosporins and carbapenems in Gram-negative bacterial pathogens. *International Journal of Medical Microbiology*.

[3] Noor R., Munna M. S. (2015). Emerging diseases in Bangladesh: Current microbiological research perspective. *Tzu Chi Medical Journal*.

[4] Levy S. (2002). *The Antibiotic Paradox: How Misuse of Antibiotics Destroys Their Curative Wers*.

[5] Kapil A. (2005). The challenge of antibiotic resistance; need to contemplate. *Indian Journal of Medical Research*.

[6] Ventola C. L. (2015). The antibiotic resistance crisis—part 1: causes and threats. *P&T*.

[7] Fair R. J., Tor Y. (2014). Antibiotics and bacterial resistance in the 21st century. *Perspectives in Medicinal Chemistry*.

[8] Prestinaci F., Pezzotti P., Pantosti A. (2015). Antimicrobial resistance: A global multifaceted phenomenon. *Pathogens and Global Health*.

[9] Friedman N. D., Temkin E., Carmeli Y. (2016). The negative impact of antibiotic resistance. *Clinical Microbiology and Infection*.

[10] Levy S. B., Marshall B. (2004). Antibacterial resistance worldwide: causes, challenges and responses. *Nature Medicine*.

[11] Mshana S. E., Matee M., Rweyemamu M. (2013). Antimicrobial resistance in human and animal pathogens in Zambia, Democratic Republic of Congo, Mozambique and Tanzania: an urgent need of a sustainable surveillance system. *Annals of Clinical Microbiology and Antimicrobials*.

[12] Karlowsky J. A., Jones M. E., Draghi D. C., Thornsberry C., Sahm D. F., Volturo G. A. (2004). Prevalence and antimicrobial susceptibilities of bacteria isolated from blood cultures of hospitalized patients in the United States in 2002. *Annals of Clinical Microbiology and Antimicrobials*.

[13] Wong C. K. M., Kung K., Au-Doung P. L. W. (2017). Antibiotic resistance rates and physician antibiotic prescription patterns of uncomplicated urinary tract infections in southern Chinese primary care. *PLoS ONE*.

[14] Walker R. D., Giguere S., Prescott J. F., Baggot J. D., Walker R. D., Dowling P. M. (2007). Antimicrobial susceptibility testing and interpretation of results. *Antimicrobial Therapy in Veterinary Medicine*.

[15] Ganesh Kumar S., Adithan C., Harish B. N., Sujatha S., Roy G., Malini A. (2013). Antimicrobial resistance in India: A review. *Journal of Natural Science, Biology and Medicine*.

[16] Johnson A. P. (2015). Surveillance of antibiotic resistance. *Philosophical Transactions of the Royal Society B: Biological Sciences*.

[17] Karam G., Chastre J., Wilcox M. H., Vincent J. (2016). Antibiotic strategies in the era of multidrug resistance. *Critical Care*.

[18] Abejew A. A., Denboba A. A., Mekonnen A. G. (2014). Prevalence and antibiotic resistance pattern of urinary tract bacterial infections in Dessie area, Northeast Ethiopia. *BMC Research Notes*.

[19] Cheesbourgh M. (2006). *District Laboratory Practice in Tropical Countries , Part II*.

[20] Mahon C., Lehman D., Manuselis G. (2011). *Iowa*.

[21] Bauer A. W., Kirby W. M., Sherris J. C., Turck M. (1966). Antibiotic susceptibility testing by a standardized single disk method. *American Journal of Clinical Pathology*.

[22] C Franklin R., Matthew A. W., Jeff A., Michael N. D., George M. E., Mary J. F. (2012). *Performance Standards for Antimicrobial Disk Susceptibility Tests*.

[23] Chander A., Shrestha C. D. (2013). Prevalence of extended spectrum beta lactamase producing Escherichia coli and Klebsiella pneumoniae urinary isolates in a tertiary care hospital in Kathmandu, Nepal. *BMC Research Notes*.

[24] Asensio A., Alvarez-Espejo T., Fernandez-Crehuet J. (2011). Trends in yearly prevalence of third-generation cephalosporin and fluoroquinolone resistant Enterobacteriaceae infections and antimicrobial use in Spanish hospitals, Spain, 1999 to 2010. *Eurosurveillance*.

[25] Polse R., Yousif S., Assafi M. (2016). Prevalence and antimicrobial susceptibility patterns of uropathogenic E. coli among people in Zakho, Iraq. *International Journal of Research in Medical Sciences*.

[26] Sabir S., Anjum A. A., Ijaz T., Ali M. A., Khan M. U. R., Nawaz M. (2014). Isolation and antibiotic susceptibility of E. coli from urinary tract infections in a tertiary care hospital. *Pakistan Journal of Medical Sciences*.

[27] Eshetie S., Tarekegn F., Kumera G., Mekonnen F. (2016). Multidrug resistant Escherichia coli strains isolated from urine sample, University of Gondar Hospital, Northwest Ethiopia. *Journal of Coastal Life Medicine*.

[28] Subha A., Ananthan S. (2002). Extended spectrum beta lactamase mediated resistance to third generation cephalosporins among Klebsiella pneumonia in Chennai. *Indian Journal medical Microbiology*.

[29] Hagi S. S., Mobaiyen H., Bayatmakoo Z. (2015). Antibiotic susceptibility of aerobic gram-negative bacilli isolated from patients admitted in intensive care units of Sina Hospital, Tabriz, Iran. *Crescent Journal of Medical and Biological Sciences*.

[30] Garbati M. A., Godhair A. I. A. (2013). The growing resistance of klebsiella pneumoniae; The need to expand our antibiogram: Case report and review of the literature. *African Journal of Infectious Diseases*.

[31] Huang S. Y., Pan K. Y., Liu X. Q. (2015). Analysis of the drug-resistant characteristics of Klebsiella pneumoniae isolated from the respiratory tract and CTX-M ESBL genes. *Genetics and Molecular Research*.

[32] Amin A., Ghumro P. B., Hussain S., Hameed A. (2009). Prevalence of antibiotic resistance among clinical isolates of Kleibsiella pneumoniaeisolated from a Tertiary Care Hospital in Pakistan. *Malaysian Journal of Microbiology*.

[33] Masood S. H., Aslam N. (2010). In vitro susceptibility test of different clinical isolates against ceftriaxone. *Oman Medical Journal*.

[34] Shoaib H. M., Baqir S. N., Sheikh D., Hashmi H. K. (2001). Cephalosporin resistance and *β*-lactamase production in clinical isolates of staphylococcus aureus in Karachi. *Pakistan Journal of Pharmaceutical Sciences*.

[35] Nkang A. O., Okonko I. O., Mejeha O. K., Adewale O. G., Udeze A. O., Fowotade A. (2009). Assessment of antibiotics susceptibility profiles of some selected clinical isolates from laboratories in Nigeria. *Journal of Microbiology and Antimicrobials*.

[36] Breurec S., Bouchiat C., Sire J.-M. (2016). High third-generation cephalosporin resistant Enterobacteriaceae prevalence rate among neonatal infections in Dakar, Senegal. *BMC Infectious Diseases*.

[37] Savas L., Guvel S., Onlen Y., Savas N., Duran N. D. (2006). Nosocomial urinary tract infections: Micro-organisms, antibiotic sensitivities and risk factors. *West Indian Medical Journal*.

[38] Khan I. U., Mirza I. A., Ikram A. (2014). Antimicrobial susceptibility pattern of bacteria isolated from patients with urinary tract infection. *Journal of the College of Physicians and Surgeons Pakistan*.

[39] Baral P., Neupane S., Marasini B. P., Ghimire K. R., Lekhak B., Shrestha B. (2012). High prevalence of multidrug resistance in bacterial uropathogens from Kathmandu, Nepal. *BMC Research Notes*.

